# Early-Life Determinants of Total and HDL Cholesterol Concentrations in 8-Year-Old Children; The PIAMA Birth Cohort Study

**DOI:** 10.1371/journal.pone.0025533

**Published:** 2011-09-27

**Authors:** Marga B.M. Bekkers, Bert Brunekreef, Henriëtte A. Smit, Marjan Kerkhof, Gerard H. Koppelman, Marieke Oldenwening, Alet H. Wijga

**Affiliations:** 1 Institute for Risk Assessment Sciences, Utrecht University, Utrecht, The Netherlands; 2 Centre for Prevention and Health Services Research, National Institute for Public Health and the Environment (RIVM), Bilthoven, The Netherlands; 3 Julius Centre for Health Sciences and Primary Care, University Medical Center Utrecht, Utrecht, The Netherlands; 4 Department of Epidemiology and Bioinformatics, University of Groningen, Groningen, The Netherlands; 5 Department of Pediatric Pulmonology and Pediatric Allergology, University Medical Center Groningen, Beatrix Children's Hospital, Groningen, The Netherlands; University of Liverpool, United Kingdom

## Abstract

**Background:**

Adult cholesterol concentrations might be influenced by early-life factors, such as breastfeeding and birth weight, referred to as “early programming”. How such early factors exert their influence over the life course is still poorly understood. Evidence from studies in children and adolescents is scarce and conflicting. We investigated the influence of 6 different perinatal risk factors on childhood total and HDL cholesterol concentrations and total-to-HDL cholesterol ratio measured at 8 years of age, and additionally we studied the role of the child's current Body Mass Index (BMI).

**Methods:**

Anthropometric measures and blood plasma samples were collected during a medical examination in 751 8-year-old children participating in the prospective Prevention and Incidence of Asthma and Mite Allergy (PIAMA) birth cohort study. Linear and logistic regression were performed to estimate associations of total and HDL cholesterol concentrations with breastfeeding, birth weight, infant weight gain, maternal overweight before pregnancy, gestational diabetes and maternal smoking during pregnancy, taking into account the child's current BMI.

**Results:**

Linear regressions showed an association between total-to-HDL cholesterol ratio and maternal pre-pregnancy overweight (β = 0.15, Confidence Interval 95% (CI): 0.02, 0.28), rapid infant weight gain (β = 0.13, 95%CI: 0.01, 0.26), and maternal smoking during pregnancy (β = 0.14, 95%CI: 0.00, 0.29). These associations were partly mediated by the child's BMI.

**Conclusion:**

Total-to-HDL cholesterol ratio in 8-year-old children was positively associated with maternal pre-pregnancy overweight, maternal smoking during pregnancy and rapid infant weight gain.

## Introduction

The risk of developing chronic disease later in life may be associated with prenatal and early postnatal factors, referred to as “early programming” [1]. Several studies showed that increased adult total cholesterol concentrations were associated with exposure to risk factors early in life [2], [3]. In a review Owen et al. [3] concluded that breastfeeding was associated with lower cholesterol concentrations in adulthood (age 17–79 years), whereas low birth weight has been associated with increased cholesterol concentrations measured at ages 17 to 76 years [4], [5], [6], [7], [8], [9].

It is of major interest whether childhood cholesterol concentrations are also associated with factors early in life, both from a public health perspective and for our understanding of the expression of early programming throughout the life course. However, only a limited number of studies have been performed on early-life factors and cholesterol concentrations in children, and their results are conflicting. Short-term breastfeeding (< 6 months) was associated with lower total cholesterol concentrations in one study [10], whereas others did not observe any difference between breastfed and non-breastfed children [11]. Higher total [12] and lower high density lipoprotein (HDL) cholesterol concentrations [13] were observed in low birth weight children, but this was not observed in two other studies [14], [15]. In children of mothers having had gestational diabetes higher total cholesterol concentrations [16] and total-to-HDL cholesterol ratio [16], and lower HDL cholesterol concentrations were observed [16], [17].

Leunissen et al. showed an inverse association between infant weight gain (first 3 months) and HDL cholesterol concentrations in young adults and a positive association with total-to-HDL cholesterol ratio [18].

We considered the joint effects of these early-life risk factors together with maternal smoking during pregnancy and maternal pre-pregnancy overweight, on childhood total and HDL cholesterol concentrations and total-to-HDL cholesterol ratio in a prospective study. To take into account the lifestyle of the child in the years preceding the cholesterol measurement, we additionally adjusted our analyses for the child's current body mass index (BMI).

## Methods

### Ethics statement

This research was performed in accordance with the ethical principles for medical research involving human subjects outlined in the Declaration of Helsinki. As 8-year-old children are considered unable to give informed consent the study protocol had to be evaluated on a national level instead of by the medical ethical committees of the participating institutes separately. Therefore, the study protocol was approved by the Dutch Central Committee on Research involving Human Subjects (P04.0071C). All parents gave written informed consent.

### Study design

The children in this study are participants of the Prevention and Incidence of Asthma and Mite Allergy (PIAMA) birth cohort study who were born in 1996–1997. A detailed description of the study design has been published previously [19]. The mothers were recruited from the general population during pregnancy visiting one of 52 prenatal clinics. Postal questionnaires were sent to the parents during pregnancy, at the child's ages of 3 and 12 months, and yearly thereafter up to the age of 8 years. At the age of 8 years a subgroup of children was invited for a hospital based medical examination.

### Study population

7,862 women were invited during pregnancy to participate in the PIAMA birth cohort study and 4,146 pregnant women agreed to participate. Of which 183 (5%) were lost to follow-up before any data of the child had been collected. Therefore the study started with 3,963 newborns. When the medical examination of 8-year-olds was planned, 3,668 children (92.6% of 3,963) were still in the study. A subgroup of 1,680 children was invited for a hospital based medical examination. Details of the subgroup selection were published before [19]. Briefly, this group consisted of 1,076 children of allergic mothers and 604 children of non-allergic mothers. Parents of 1263 children (833 of allergic mothers and 430 of non allergic mothers) gave informed consent for the child to participate in the medical examination and 1132 children (748 of allergic mothers and 384 of non-allergic mothers) attended the hospital based medical examination. From 817 children (524 of allergic mothers and 293 of non allergic mothers) (non-fasted) a blood sample for cholesterol measurement was obtained and in 790 of these samples (505 of children of allergic mothers and 285 of children of non allergic mothers) cholesterol concentrations could be determined. For the present analyses 3 children were excluded because of missing data for gestational age. All children (n = 36) with a gestational age of less than 37 weeks were excluded from the analysis to avoid possible confounding by prematurity. The final study population consisted of 751 children.

Children with missing data on infant weight gain (n = 2), maternal BMI before pregnancy (n = 51), gestational diabetes (n = 12), or smoking of the mother during pregnancy (n = 9) but with data on cholesterol concentrations, were not excluded from all analyses, but only from the analysis of the specific risk factor for which a value was missing.

No differences in total and HDL cholesterol concentrations and total-to-HDL cholesterol ratio were observed between children with a missing value on one of the risk factors and children with complete data on all risk factors.

### Exposure and outcome variables

Plasma total and HDL cholesterol concentrations were determined enzymatically using Roche automated clinical chemistry analyzers (Roche Diagnostics, Indianapolis). [20] Additionally the ratio between total and HDL cholesterol was calculated (total-to-HDL cholesterol ratio).

The exposure variables birth weight, breastfeeding, BMI of the mother before pregnancy, infant weight gain, gestational diabetes of the mother during pregnancy of the index child, and smoking of the mother during pregnancy were reported by the parents in the questionnaires. To study both the effects of being born with a low or a high birth weight, birth weight categories were defined as low birth weight (< 2,500g), birth weight ≥2,500g and <4,000g, and high birth weight (≥4,000g). Duration of breastfeeding was assessed by questions on infant feeding in the questionnaires at 3 months and 1 year of age. Breastfeeding was defined as any kind of breastfeeding, including partial breastfeeding. Total breastfeeding duration was categorized in no breastfeeding, breastfeeding 1 to 16 weeks, or ≥16 weeks of breastfeeding. Maternal BMI before pregnancy was calculated using pre-pregnancy body weight and height reported in the 1 year questionnaire, and overweight was defined based on international defined cut-off points, i.e. >25kg/m^2^. Infant weight gain during the first year of life was calculated using data on body weight reported by the parents in the first year. The parents were asked to report the body weight as was measured by the health centre, which is visited regularly during the first years of life of a child. Infant weight gain was then divided into tertiles (slow <6.13 kg/year, intermediate ≥6.14kg/year and <6.98kg/year and rapid ≥6.99kg/year weight gain). The child's BMI at age 8 years was calculated using body weight and height measured at the time of blood sampling. Body weight was measured at the nearest 0.1kg and height (cm) was measured at one decimal, both anthropometric variables were measured while only wearing underwear. BMI for age Standard Deviation Scores (SDS) were calculated using the reference growth curves of the Dutch Fourth Nation-wide Growth Study carried out in 1997 [21].

### Statistical analyses

For all statistical analyses SAS software version 9.2 (SAS Institute, Inc., Cary, NC) was used. First means and standard deviations of total and HDL cholesterol concentrations, and total-to-HDL cholesterol ratio were calculated. Bell et al. [22] suggested to consider children with total cholesterol concentrations higher than 5.9 mmol/l, as well as children with HDL cholesterol concentrations below 0.8 mmol/l at risk of coronary heart diseases. As the prevalence of total cholesterol concentrations >5.9 mmol/l in this study population was low, the cholesterol concentrations were divided into tertiles for the analyses.

Simple and multivariate linear regression were performed to estimate the associations of total and HDL cholesterol, and total-to-HDL cholesterol ratio with the early life factors. Logistic regression was performed with total cholesterol concentrations or total-to-HDL cholesterol ratio in the highest tertile of the distribution compared with the lowest tertile, and with HDL cholesterol concentrations in the lowest tertile of the distribution, compared to the highest tertile as binary outcome variables.

All analyses were performed with and without adjustment for potential confounders. A first model included age of the child (in days) at time of blood sampling and gender. A more detailed model was additionally adjusted for gestational age, maternal education (low, intermediate, high education), and the other exposure variables. Additionally, the child's BMI SDS was added to the fully adjusted model to investigate to which extent the associations between early-life determinants and cholesterol concentrations were explained by BMI as an indicator of the child's lifestyle.

Additionally the regression analyses were performed separately for boys and girls, and separately for children of allergic and non-allergic mothers to investigate the presence of effect modification by these variables.

## Results

**Figure 1:**
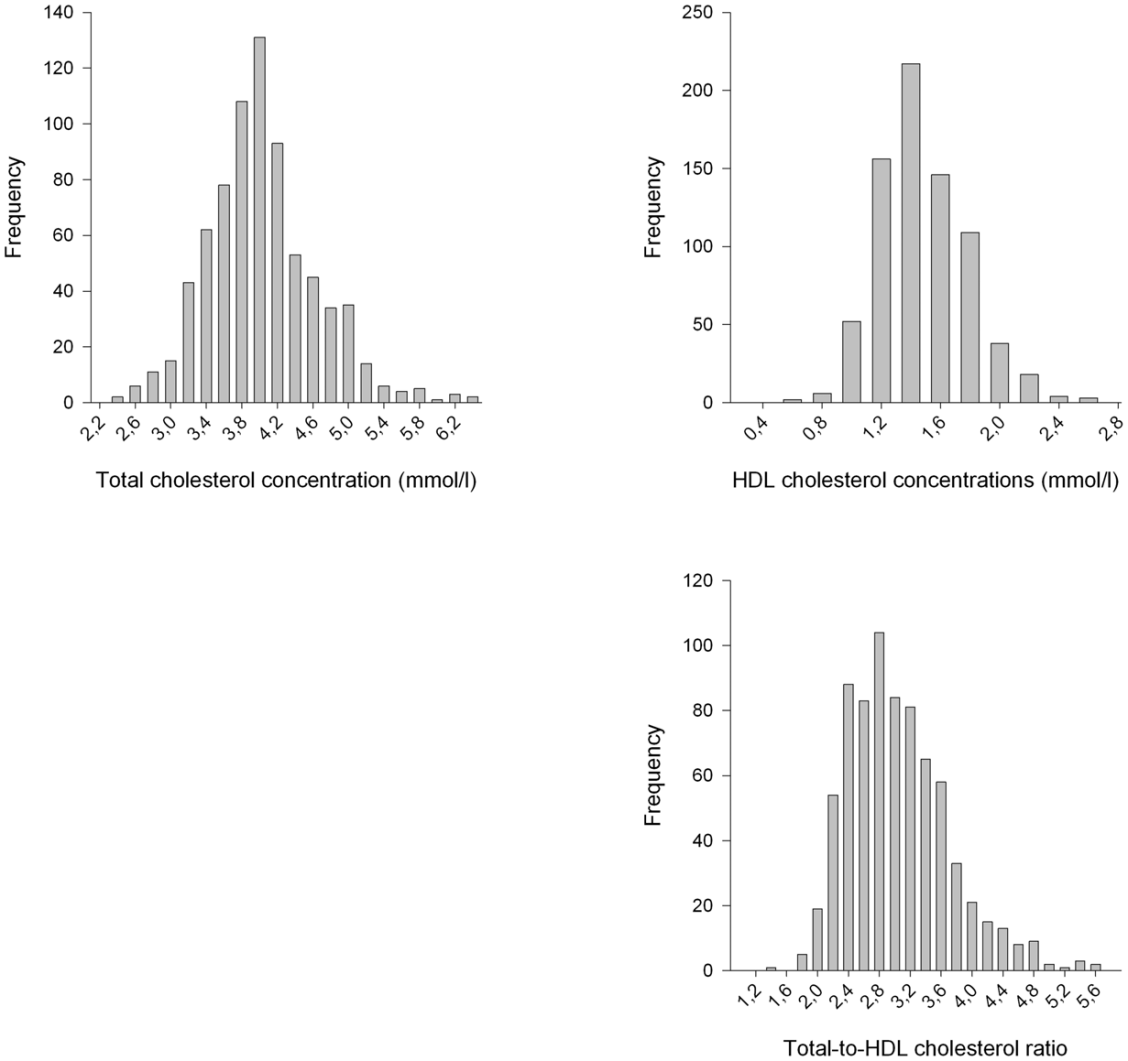
Distribution of cholesterol variables.

The total mean cholesterol concentration ranged from 2.37 to 6.32 mmol/L in girls and from 2.26 to 6.27 mmol/L in boys (Figure 1). Children of mothers with gestational diabetes had lower mean total cholesterol concentrations (3.81 vs. 3.93 mmol/L), but only 12 mothers (1.6%) had gestational diabetes and the association was non-significant (Table 1).

**Table 1:** Mean values and standard deviations (std) of total and HDL cholesterol concentrations and total-to-HDL cholesterol ratio.

			Total cholesterol mmol/L	HDL cholesterol mmol/L	Total-to-HDL cholesterol ratio
		n	Mean ± std	Mean ± std	Mean ± std
**Total Group**		751	3.93±0.62	1.38±0.30	2.95±0.70
**Gender**					
	Boys	384	3.85±0.63*	1.40±0.31	2.85±0.64*
	Girls	367	4.01±0.60	1.36±0.30	3.06±0.74
**Breastfeeding**					
	None	112	3.95±0.58	1.39±0.31	2.95±0.69
	< 16 weeks	349	3.94±0.60	1.38±0.29	2.96±0.67
	≥16 weeks	290	3.92±0.65	1.38±0.31	2.95±0.73
**Birth weight**					
	< 2,500g	15	3.92±0.62	1.37±0.28	3.00±0.96
	>2,500g and <4,000g	587	3.94±0.61	1.38±0.31	2.96±0.70
	≥4,000g	149	3.89±0.66	1.37±0.30	2.93±0.64
**Infant weight gain (n = 649)**					
	< 6.13 kg/year	216	3.94±0.60	1.37±0.28	2.97±0.71
	≥6.14 and <6.98kg/year	217	3.89±0.61	1.39±0.28	2.90±0.65
	≥6.99 kg/year	216	3.92±0.62	1.39±0.34	2.94±0.68
**Overweight mother before pregnancy**					
	Yes	143	3.93±0.63	1.35±0.30	3.06±0.88
	No	557	3.93±0.63	1.39±0.30	2.92±0.64
**Gestational diabetes**					
	Yes	12	3.81±0.58	1.38±0.34	2.87±0.53
	No	727	3.93±0.62	1.38±0.30	2.95±0.70
**Smoking mother during pregnancy**					
	Yes	114	3.96±0.67	1.33±0.30	3.10±0.78**
	No	628	3.93±0.61	1.39±0.30	2.93±0.68

*P<0.01.

**P<0.05.

**Figure 2:**
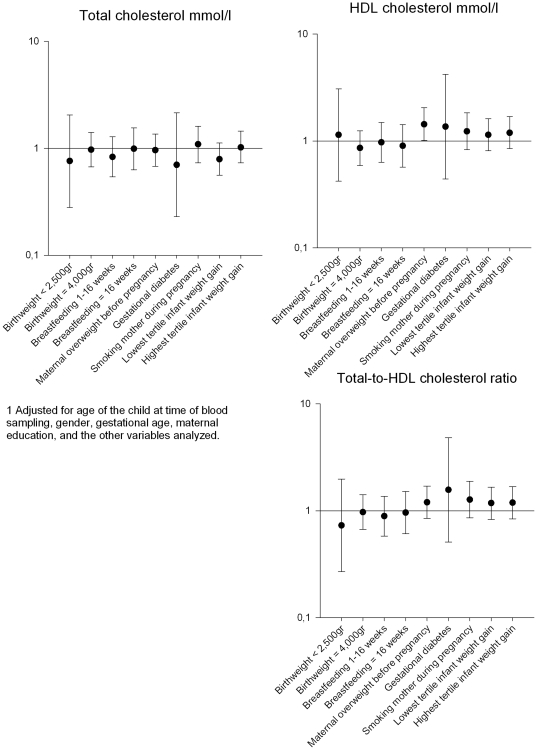
Adjusted^1^ odds ratios with 95% confidence intervals.

Maternal pre-pregnancy overweight and rapid infant weight gain were, in the fully adjusted models, associated with an increase in total-to-HDL cholesterol ratio of 0.15 (95% Confidence Interval (CI) 0.02 to 0.28) and 0.13 (95%CI 0.01 to 0.26) respectively (Table 2). Maternal smoking was associated with an increase in total-to-HDL cholesterol ratio of 0.14 (95%CI 0.00 to 0.29). In logistic regression no significant associations of the early life risk factors with the tertiles of total and HDL cholesterol, and total-to-HDL cholesterol ratio were found (Figure 2). The results of maternal overweight before pregnancy were borderline similar to those of the linear regression analyses.

**Table 2:** Associations between total and HDL cholesterol, and total-to-HDL cholesterol ratio and early life factors for 751 Dutch children ^a^.

	Total cholesterol		HDL cholesterol		Total-to-HDL cholesterol ratio	
	β	95% CI	β	95% CI	β	95% CI
**Birth weight <2500g**						
Crude model	−0.02	−0.33, 0.29	−0.01	−0.17, 0.15	0.04	−0.33, 0.40
Adjusted model A b	−0.04	−0.35, 0.27	−0.01	−0.16, 0.15	0.02	−0.34, 0.38
Adjusted model B c	−0.05	−0.38, 0.28	−0.04	−0.20, 0.12	0.08	−0.29, 0.46
**Birth weight >4000g**						
Crude model	−0.06	−0.17, 0.06	−0.01	−0.07, 0.04	−0.02	−0.15, 0.10
Adjusted model A b	−0.04	−0.15, 0.07	−0.02	−0.07, 0.04	−0.003	−0.12, 0.13
Adjusted model B c	−0.001	−0.13, 0.13	0.01	−0.05, 0.07	−0.03	−0.17, 0.07
**Breastfeeding 1**−**16wk**						
Crude model	−0.01	−0.13, 0.12	−0.01	−0.08, 0.05	0.02	−0.13, 0.16
Adjusted model A b	−0.01	−0.14, 0.12	−0.01	−0.08, 0.05	0.01	−0.13, 0.15
Adjusted model B c	−0.01	−0.15, 0.13	−0.01	−0.08, 0.06	0.03	−0.13, 0.18
**Breastfeeding** ≥**16wk**						
Crude model	−0.03	−0.17, 0.11	−0.01	−0.08, 0.06	0.004	−0.15, 0.16
Adjusted model A b	−0.03	−0.17, 0.10	−0.01	−0.08, 0.06	−0.002	−0.16, 0.15
Adjusted model B c	0.003	−0.15, 0.16	−0.002	−0.08, 0.07	0.04	−0.13, 0.22
**Maternal overweight before pregnancy**						
Crude model	0.004	−0.11, 0.12	−0.04	−0.10, 0.01	0.13	0.005, 0.26*
Adjusted model A b	−0.01	−0.11, 0.12	−0.04	−0.10, 0.01	0.13	0.01, 0.26*
Adjusted model B c	0.01	−0.10, 0.13	−0.05	−0.10, 0.01	0.15	0.02, 0.28*
**Gestational diabetes**						
Crude model	−0.12	−0.47, 0.24	−0.01	−0.18, 0.17	−0.08	−0.48, 0.31
Adjusted model A b	−0.10	−0.46, 0.25	−0.01	−0.18, 0.16	−0.07	−0.46, 0.32
Adjusted model B c	−0.19	−0.57, 0.19	−0.06	−0.24, 0.13	−0.07	−0.49, 0.35
**Smoking mother during pregnancy**						
Crude model	0.04	−0.09, 0.16	−0.06	−0.12, 0.003	0.17	0.02, 0.31*
Adjusted model A b	0.02	−0.10, 0.14	−0.05	−0.11, 0.01	0.15	0.01, 0.29*
Adjusted model B c	0.01	−0.13, 0.14	−0.06	−0.12, 0.01	0.14	−0.001, 0.29
**Slow infant weight gain**						
Crude model	0.08	−0.04, 0.19	−0.004	−0.06, 0.05	0.09	−0.04, 0.22
Adjusted model A b	0.06	−0.06, 0.17	0.003	−0.05, 0.06	0.06	−0.07, 0.18
Adjusted model B c	0.06	−0.07, 0.18	0.01	−0.05, 0.06	0.07	−0.07, 0.20
**Rapid infant weight gain**						
Crude model	0.06	−0.05, 0.18	−0.01	−0.07, 0.04	0.11	−0.01, 0.23
Adjusted model A b	0.09	−0.02, 0.20	−0.02	−0.08, 0.04	0.15	0.03, 0.27*
Adjusted model B c	0.10	−0.02, 0.22	−0.01	−0.07, 0.05	0.13	0.01, 0.26*

*p<0.05.

a) Results are presented as regression coefficients (β) with 95% confidence interval (CI).

b) Regression model A is adjusted for age of the child at time of blood sampling and gender.

c) Regression model B is adjusted for age of the child at time of blood sampling, gender, gestational age, maternal education, and the other variables analyzed.

Children of mothers who were overweight, children of mothers who smoked during pregnancy, and children who gained weight rapidly during infancy all had higher BMI SDS at the age of 8 years than their counterparts (0.47 *vs.* −0.03 (*P*<.0001), 0.22 *vs.* 0.04 (*P* = .05), 0.40 *vs.* 0.10 (*P* = .0004), respectively). Higher BMI SDS values, in turn, were associated with higher total-to-HDL cholesterol ratio (β = 0.13 (95%CI 0.08 to 0.18). When BMI SDS was added to the fully adjusted regression models (Model B in Table 2) the effect estimates decreased to 0.09 (95%CI −0.04 to 0.22) for maternal pre-pregnancy overweight, 0.12 (95%CI −0.02 to 0.27) for maternal smoking during pregnancy, and 0.09 (95%CI −0.04 to 0.22) for rapid infant weight gain. Current BMI thus partly, but not entirely, explained the association between total-to-HDL cholesterol ratio and maternal overweight before pregnancy, maternal smoking during pregnancy, and rapid infant weight.

In stratified analyses no differences in associations between cholesterol concentrations and the exposure variables between boys and girls and between children of allergic and non-allergic mothers were found.

## Discussion

The results of the present study suggest an association between maternal pre-pregnancy overweight, maternal smoking during pregnancy and rapid infant weight gain and total-to-HDL cholesterol ratio. These associations were attenuated after adjustment for BMI of the child at the time of blood sampling.

### Strengths and limitations

The strengths of our study are its prospective design, the possibility to study a range of perinatal and early life factors, the large size of the study population compared to most previous studies, the ability to adjust the different factors for each other and for BMI SDS at the time of the cholesterol measurement, and the availability of information on several confounding factors.

However, our study also has some limitations; the number of mothers having had gestational diabetes, and the number of children born *full term* with a low birth weight (<2,500g) were small, which may have limited the study's potential to detect associations with these factors. Data on the risk factors studied were obtained from parental reports and the possibility of reporting errors cannot be excluded. However, the data on the early life factors were obtained prospectively, consequently we do not expect recall bias. Furthermore, there is no indication that other types of misreporting in association with cholesterol concentrations explained our results. Only for a subset of the baseline population blood samples were available. There is no reason to assume that associations differ between children with and without data on cholesterol concentrations. In addition, children of allergic mothers were overrepresented, but no effect modification by maternal allergy was observed. Because of the design of the medical examination, it was not possible to take blood samples of children in the fasted state. In The PIAMA birth cohort study low educated mothers and children from ethnic minorities are under represented compared with the general Dutch population, however, we do not expect that these differences would have consequences for the generalisability of our results.

### Findings of other studies

We compared the cholesterol concentrations measured in our study to those measured in other child populations. The total and HDL cholesterol concentrations in our study population were similar to those measured in comparable childhood populations [10], [14], [23], although only one study also showed significantly higher total cholesterol concentrations in girls than in boys [10]. Only one previous study reported the mean total-to-HDL cholesterol ratio found in their childhood population [24]. The mean total-to-HDL cholesterol ratio in our study population was somewhat higher compared to this recent study in 9-year-old UK children.

Studies on associations between cholesterol concentrations and early life risk factors in adults have mainly focused on breastfeeding and low birth weight. In a review including 17 original studies, slightly lower cholesterol concentrations were found in adults who had been breastfed [3], [4], [5], [7], [25], [26]. Low birth weight has only been associated with increased total cholesterol concentrations in men (age 17–76 years old) [2], [4], [5], [7], [9]. In women (17–64 years old), no association between low birth weight and cholesterol concentrations has been reported [5], [27].

So far, there have been few studies on the association between breastfeeding and birth weight and cholesterol concentrations in children. Higher total cholesterol concentrations [12] and lower HDL cholesterol concentrations [13] were found in low birth weight children, but this was not found in two other studies (Table 3) [14], [15]. Bergstrom et al. [10] observed lower total cholesterol concentrations in breastfed children, whereas Fomon et al. [11] did not observe any difference between breastfed and non-breastfed children. Higher total cholesterol concentrations [16] and total-to-HDL cholesterol ratios [16] and lower HDL cholesterol concentrations [16], [17] were observed in children of mothers having had gestational diabetes.

**Table 3:** Studies on early factors influencing childhood cholesterol levels.

Author	Year of publication	Number of subjects	Study design	Age (years)	Number of girls	Early factors studied	Adjustment for confounders	Total cholesterol	HDL cholesterol	Total-to-HDL cholesterol ratio
Bergström et al.	1995	405	Prospective	14	201 (49.6%)	Breastfeeding <6 months	Nof	*r* = -0.07, *P* = 0.057		
Fomon et al.	1984	469	Prospective	8	188 (40.1%)	Any breastfeeding	No	Males: *r* = 0.393 NS Females: *r* = 0.232 NS		
Frontini et al.	2004	1141	Retrospective	4–11	536 (47%)	Low birth weight	Age, race, sex		Lower mean HDL cholesterol (p = 0.05)	
Leunissen et al.	2008	297	Retrospective	18–24	181 (60.9%)	Low birth weight (birth weight sds)	No	β = nm, *P* = ns	β = nm, *P* = ns	
Leunissen et al.	2009	205	Retrospective	18–24	Nm	Weight gain first 3 months of life	Gestational age, sex, age, ses, height growth	β = −0.002, *P* = .89	β = −0.053, *P* = .005	β = 0.052, *P* = .01
Manderson et al.	2002	118	Retrospective	5–11	65 (55.1%)	Gestational diabetes	No	Diff non gestdiab-gestdiab: 0.27, *P* = 0.03	Diff non gestdiab-gestdiab: −0.05, *P* = 0.38	Diff non gestdiab-gestdiab: 0.33, *P* = 0.03
Mortaz et al.	2001	412	Prospective	8–12	197 (47.8%)	Birth weight <1,850g	No	β = nm, *P* = ns		
Tenhola et al.	2000	110	Prospective	12	70 (63.6%)	Small for gestational age	No	SGA higher TC concentration	NS diff between SGA and AGA children	
Tam et al.	2008	164	retrospective	7–10	83 (50.6%)	Gestational diabetes	Age, sex		Lower HDL level (*P* = 0.019)	

Legend: Nm = Not mentioned, r = correlation coefficient, NS =  non-significant, SGA =  small for gestational age, AGA = appropriate for gestational age, TC = total cholesterol, SES = socio-economic status.

We were able to study the associations with other potential early–life determinants that were not studied earlier in adults or children. We observed associations between maternal pre-pregnancy overweight, rapid infant weight gain and maternal smoking during pregnancy and total-to-HDL cholesterol ratio in our study. These associations attenuated after adjustment for the child's BMI SDS. The interpretation is not straightforward: early-life determinants apparently influence both BMI and cholesterol at age 8. The lifestyle of the child in the years preceding the cholesterol measurement plays an important role in the associations between early-life determinants and childhood cholesterol concentrations and risk factors that occur during the intervening years must be considered. Possibly also genetic factors contribute to these associations. We adjusted the associations between the early-life determinants and childhood cholesterol concentrations for BMI SDS as a summary indicator of the child's lifestyle in the years preceding the cholesterol measurement, to assess to which extent associations these associations were independent of lifestyle.

The observed effects of the investigated early life risk factors are small, but relatively small changes in the cholesterol profile may have an impact on the burden of chronic diseases later in life [28]. If confirmed in future studies, these early-life determinants might be a lead for prevention.

### Conclusion

Total-to-HDL cholesterol ratio in 8-year-old children was positively associated with maternal pre-pregnancy overweight, maternal smoking during pregnancy and rapid infant weight gain.

## References

[1] Wells JC, Chomtho S, Fewtrell MS (2007). Programming of body composition by early growth and nutrition.. Proc Nutr Soc.

[2] Lawlor DA, Owen CG, Davies AA, Whincup PH, Ebrahim S (2006). Sex differences in the association between birth weight and total cholesterol. A meta-analysis.. Ann Epidemiol.

[3] Owen CG, Whincup PH, Kaye SJ, Martin RM, Davey Smith G (2008). Does initial breastfeeding lead to lower blood cholesterol in adult life? A quantitative review of the evidence.. Am J Clin Nutr.

[4] Barker DJ, Martyn CN, Osmond C, Hales CN, Fall CH (1993). Growth in utero and serum cholesterol concentrations in adult life.. Bmj.

[5] Davies AA, Smith GD, Ben-Shlomo Y, Litchfield P (2004). Low birth weight is associated with higher adult total cholesterol concentration in men: findings from an occupational cohort of 25,843 employees.. Circulation.

[6] Lawlor DA, Ronalds G, Clark H, Smith GD, Leon DA (2005). Birth weight is inversely associated with incident coronary heart disease and stroke among individuals born in the 1950s: findings from the Aberdeen Children of the 1950s prospective cohort study.. Circulation.

[7] Martyn CN, Gale CR, Jespersen S, Sherriff SB (1998). Impaired fetal growth and atherosclerosis of carotid and peripheral arteries.. Lancet.

[8] Owen CG, Whincup PH, Odoki K, Gilg JA, Cook DG (2003). Birth weight and blood cholesterol level: a study in adolescents and systematic review.. Pediatrics.

[9] Huxley R, Owen CG, Whincup PH, Cook DG, Colman S (2004). Birth weight and subsequent cholesterol levels: exploration of the “fetal origins” hypothesis.. Jama.

[10] Bergstrom E, Hernell O, Persson LA, Vessby B (1995). Serum lipid values in adolescents are related to family history, infant feeding, and physical growth.. Atherosclerosis.

[11] Fomon SJ, Rogers RR, Ziegler EE, Nelson SE, Thomas LN (1984). Indices of fatness and serum cholesterol at age eight years in relation to feeding and growth during early infancy.. Pediatr Res.

[12] Tenhola S, Martikainen A, Rahiala E, Herrgard E, Halonen P (2000). Serum lipid concentrations and growth characteristics in 12-year-old children born small for gestational age.. Pediatr Res.

[13] Frontini MG, Srinivasan SR, Xu J, Berenson GS (2004). Low birth weight and longitudinal trends of cardiovascular risk factor variables from childhood to adolescence: the bogalusa heart study.. BMC Pediatr.

[14] Mortaz M, Fewtrell MS, Cole TJ, Lucas A (2001). Birth weight, subsequent growth, and cholesterol metabolism in children 8-12 years old born preterm.. Arch Dis Child.

[15] Leunissen RW, Kerkhof GF, Stijnen T, Hokken-Koelega AC (2008). Fat mass and apolipoprotein E genotype influence serum lipoprotein levels in early adulthood, whereas birth size does not.. J Clin Endocrinol Metab.

[16] Manderson JG, Mullan B, Patterson CC, Hadden DR, Traub AI (2002). Cardiovascular and metabolic abnormalities in the offspring of diabetic pregnancy.. Diabetologia.

[17] Tam WH, Ma RC, Yang X, Ko GT, Tong PC (2008). Glucose intolerance and cardiometabolic risk in children exposed to maternal gestational diabetes mellitus in utero.. Pediatrics.

[18] Leunissen RW, Kerkhof GF, Stijnen T, Hokken-Koelega A (2009). Timing and tempo of first-year rapid growth in relation to cardiovascular and metabolic risk profile in early adulthood.. Jama.

[19] Brunekreef B, Smit J, de Jongste J, Neijens H, Gerritsen J (2002). The prevention and incidence of asthma and mite allergy (PIAMA) birth cohort study: design and first results.. Pediatr Allergy Immunol.

[20] Cloey T, Bachorik PS, Becker D, Finney C, Lowry D (1990). Reevaluation of serum-plasma differences in total cholesterol concentration.. Jama.

[21] Fredriks AM, van Buuren S, Burgmeijer RJ, Meulmeester JF, Beuker RJ (2000). Continuing positive secular growth change in The Netherlands 1955-1997.. Pediatr Res.

[22] Bell RD, Macek M, Rutenfranz GJ, Saris HM, Rutenfranz GJea (1986). Health factors and risk indicators of cardiovascular diseases during childhood and adolescence.. Children and exercise XII: Human Kinetics.

[23] Thorsdottir I, Gunnarsdottir I, Palsson GI (2003). Association of birth weight and breast-feeding with coronary heart disease risk factors at the age of 6 years.. Nutr Metab Cardiovasc Dis.

[24] Gardner DS, Hosking J, Metcalf BS, Jeffery AN, Voss LD (2009). Contribution of early weight gain to childhood overweight and metabolic health: a longitudinal study (EarlyBird 36).. Pediatrics.

[25] Ravelli AC, van der Meulen JH, Osmond C, Barker DJ, Bleker OP (2000). Infant feeding and adult glucose tolerance, lipid profile, blood pressure, and obesity.. Arch Dis Child.

[26] Schack-Nielsen L, Michaelsen KF (2006). Breast feeding and future health.. Curr Opin Clin Nutr Metab Care.

[27] Mogren I, Hogberg U, Stegmayr B, Lindahl B, Stenlund H (2001). Fetal exposure, heredity and risk indicators for cardiovascular disease in a Swedish welfare cohort.. Int J Epidemiol.

[28] Lewington S, Whitlock G, Clarke R, Sherliker P, Emberson J (2007). Blood cholesterol and vascular mortality by age, sex, and blood pressure: a meta-analysis of individual data from 61 prospective studies with 55,000 vascular deaths.. Lancet.

